# Navicular bone morphometry for implant planning in fracture surgery: a dry bone analysis

**DOI:** 10.1007/s00264-026-06889-x

**Published:** 2026-06-06

**Authors:** Helin Yücedağ Gündoğdu, Bahattin Paslı

**Affiliations:** Hacettepe University, Ankara, Turkey

**Keywords:** Navicular bone, Osseous anatomy, Navicular fracture, Internal fixation, Safe screw trajectory, Reconstructive planning

## Abstract

**Objective:**

The purpose of this study is to quantitatively characterise surgically relevant morphometric features of dry navicular bones and to provide anatomical reference values that can be utilised in implant placement.

**Methods:**

Fifty-five isolated, unpaired dry navicular bones without macroscopic deformity were examined. Tuberosity morphology, tuberosity–medial cuneiform distances, navicular body dimensions (mediolateral width, level-specific anteroposterior measurements, and depth), talonavicular articular surface depth, and concavity index were evaluated using a digital caliper and digital thickness gauge.

**Results:**

The narrowest tuberosity width, anteroposterior length, and supero-inferior height were 6.59 ± 1.28 mm, 16.38 ± 2.43 mm, and 11.24 ± 1.26 mm, respectively. The tuberosity groove depth was 2.05 ± 0.92 mm. The distances from the tuberosity midpoint to the medial cuneiform articular surface at the nearest and farthest points were 21.86 ± 3.11 mm, 29.65 ± 3.37 mm. The mean mediolateral body width was 37.69 ± 3.54 mm. Level-specific anteroposterior dimensions were 18.01 ± 2.34 mm, 15.15 ± 2.15 mm, and 12.56 ± 1.62 mm, whereas the corresponding body depths were 18.35 ± 3.01 mm, 25.24 ± 2.30 mm, and 22.52 ± 2.47 mm, respectively.

**Conclusion:**

These findings provide surgically relevant anatomical reference values that may assist preoperative planning and support safer fixation of navicular fractures, and contribute to the design and optimisation of navicular implants and fixation devices.

## Introduction

The tarsal navicular bone is a *keystone* that plays a pivotal role in load transfer between the talus and distal tarsal bones, as well as in maintaining the medial longitudinal arch. Due to its anatomical location and multiple joint relationships, disruptions to the integrity of this structure can directly affect midfoot biomechanics and medial column stability. The primary clinical objective in navicular injuries is therefore to ensure stability in a manner that preserves foot function and maintains the integrity of weight-bearing structures [1, 2].

Although tarsal navicular fractures are rare, injuries in this region have been reported to account for approximately 0.45% of all fractures, 5.1% of foot fractures, and 35% of midfoot fractures [3, 4]. These injuries are generally classified as traumatic and stress fractures. The Sangeorzan classification is a widely utilised system, particularly in the evaluation of navicular body fractures [5]. The treatment approach is determined according to the degree of displacement, joint congruity, and medial column stability. While conservative treatment is preferred for small and stable fractures, reduction and stable fixation are frequently required for displaced or comminuted body fractures [1, 2]. Surgical treatment options encompass open reduction and internal fixation (ORIF), in addition to closed or percutaneous techniques. However, the complex anatomy of the navicular and the limited osseous corridor available for fixation render implant placement technically challenging [1, 2, 4, 6].

Additionally, the vascular anatomy of the navicular can also affect stress fracture development and fracture healing. Cadaveric studies have shown areas of reduced intraosseous vascularity overlapping with areas where stress fractures are frequently observed [1, 7, 8]. These biological features increase the risk of delayed union, nonunion, and avascular necrosis, highlighting the importance of preserving not only mechanical stability but also bone biology in treatment planning. Furthermore, the complex three-dimensional morphology of the navicular bone may present challenges for implant adaptation and fixation. Therefore, detailed morphometric data may contribute not only to safer fixation planning but also to the development of anatomically compatible implants for navicular surgery.

Despite the clinical importance of navicular fractures, level-specific osseous data relevant to implant placement remain limited. This may complicate preoperative planning and implant selection in navicular fracture surgery. Therefore, this study aimed to provide surgically relevant morphometric reference values from dry navicular bones for fixation planning.

## Materials and methods

### Study design

This descriptive morphometric study included 55 isolated, unpaired dry navicular bones with preserved anatomical integrity and no macroscopic deformity. The age and sex of the subjects are unavailable. Prior to measurement, all bones were positioned in a standard anatomical orientation, using the talonavicular articular surface and tuberosity as reference points.

This study was conducted on a collection of dry navicular bones obtained from the Department of Anatomy, Faculty of Medicine Hacettepe University. The study protocol was approved by the Ethics Committee of the Faculty of Medicine, Hacettepe University (Approval No: 2025/17–28).

### Parameters

**Fig. 1:**
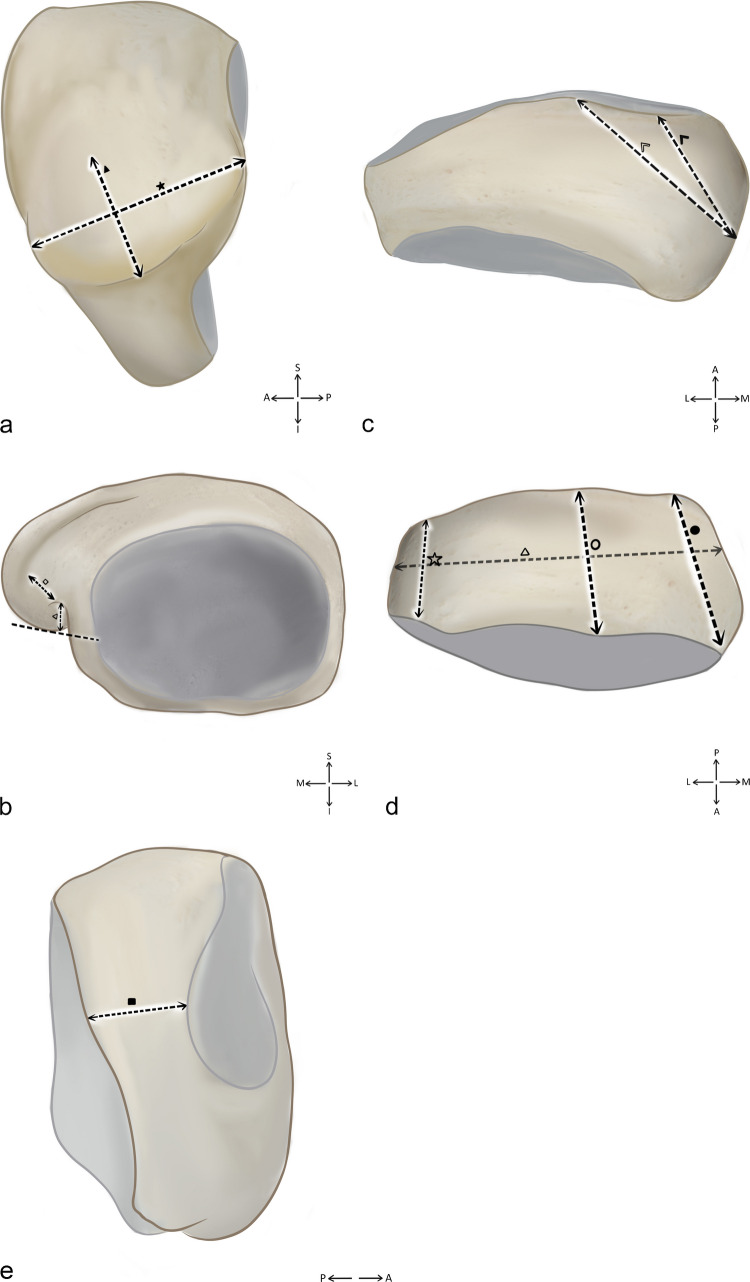
Schematic illustration of the morphometric parameters. (**a, b**) Navicular tuberosity measurements: ***black filled star–triangle***, anteroposterior length and supero-inferior height; ***black unfilled square,*** minimum mediolateral width; ***black unfilled triangle,*** depth of the tibialis posterior tendon groove. (**c**) ***Black filled–unfilled arrowhead,*** distance from the midpoint of the tuberosity to the medial cuneiform articular surface at the nearest and farthest points. (**d**) Dorsal surface measurements of the navicular body: ***black filled circle,*** anteroposterior dimension at the beginning of the medial cuneiform articular surface; ***black unfilled circle,*** anteroposterior dimension at the end of the medial cuneiform articular surface; ***black unfilled star,*** anteroposterior dimension at the terminal level of the dorsal surface; ***black unfilled triangle,*** mediolateral width. (**e**) ***Black filled square,*** anteroposterior dimension of the lateral surface. Orientation arrows indicate anatomical directions: superior (S), inferior (I), anterior (A), posterior (P), medial (M), lateral (L), as appropriate

Linear morphometric measurements were performed to define osseous morphology and assess regional bone stock relevant to surgical fixation. Distances were measured with a digital caliper with 0.01 mm accuracy, and thickness was measured with an outside diameter digital thickness gauge, using predefined anatomical reference points. All measurements were obtained in accordance with a standardised protocol. Tuberosity morphology was assessed by minimum mediolateral width, anteroposterior length, supero-inferior height, and depth of the tibialis posterior tendon groove (Fig. 1a-1b). The distance from the midpoint of the tuberosity to the medial cuneiform articular surface was measured at its nearest and farthest points (Fig. 1c). Body measurements were performed using the dorsal surface as a reference. Mediolateral width was measured once, whereas anteroposterior dimensions and body depth were recorded at the beginning and end of the medial cuneiform articular surface and at the terminal level of the dorsal surface (Fig. 1d). In addition, anteroposterior dimension of the lateral surface was recorded (Fig. 1e). For the proximal articular surface articulating with the talar head, articular surface depth and concavity index were measured. The concavity index was calculated by dividing the perpendicular distance from the midpoint of the chord to the deepest point of the articular surface by chord length between the medial and lateral edges of the articular surface.

To minimise measurement errors, each parameter was assessed by two independent observers. The reliability of the measurements was determined using the intraclass correlation coefficient (ICC), with ICC > 0.90 considered high reliability.

### Statistical analysis

Statistical analyses were performed using SPSS v23.0 (IBM Corp., Armonk, NY, USA). Continuous variables were expressed as mean, standard deviation, percentiles and minimum–maximum values. The assessment of normality was undertaken utilising the Shapiro–Wilk test and histograms. Level-specific measurements from the same bone were compared using the Friedman test. When significant, pairwise comparisons were performed with the Wilcoxon signed-rank test. Furthermore, the Bonferroni correction was applied for multiple comparisons, and the significance level was accepted as *p* < 0.0167, whereas *p* < 0.05 was used for all other analyses.

## Results

A total of 55 dry navicular bones, including 25 on the right and 30 on the left sides, were evaluated. Despite the detection of statistically significant variations between the right and left sides in some parameters, the results are presented on the basis of the total sample due to the inability to confirm that the samples examined were actually matched bones belonging to the same individuals.

### Tuberosity morphology

The narrowest width of the navicular tuberosity was 6.59 ± 1.28 mm (range; 2.90–9.77 mm). Its anteroposterior length and supero-inferior height were 16.38 ± 2.43 mm (range; 11.83–21.72 mm) and 11.24 ± 1.26 mm (range; 8.86–14.40 mm), respectively. The depth of the tibialis posterior tendon groove was 2.05 ± 0.92 mm (range; 0.03–4.71 mm). The distances from the midpoint of the tuberosity to the medial cuneiform articular surface were 21.86 ± 3.11 mm (range; 15.56–30.85 mm) at the nearest point and 29.65 ± 3.37 mm (range; 23.33–36.44 mm) at the farthest point (Table 1).

**Table 1:** Talonavicular joint surface depth, navicular tuberosity measurements and distances to the medial cuneiform articular surface

Parameter			Mean ± SD (mm)	10% – 90% percentile	Range
Talonavicular joint surface	Depth		5.27 ± 0.92	4.02–6.58	3.03–7.17
Tuberosity measurements		Narrowest width	6.59 ± 1.28	5.17–8.05	2.90–9.77
		Anteroposterior length	16.38 ± 2.43	13.44–20.17	11.83–21.72
		Supero-inferior height	11.24 ± 1.26	9.56–12.88	8.86–14.40
Depth of the tibialis posterior tendon groove			2.05 ± 0.92	0.78–3.25	0.03–4.71
Tuberosity midpoint–MCAS		at the nearest point	21.86 ± 3.11	18.10–25.66	15.56–30.85
		at the farthest point	29.65 ± 3.37	25.74–34.51	23.33–36.44

*SD* standard deviation, *MCAS* medial cuneiform articular surface

### Navicular body dimensions

The mediolateral width of the body was determined to be 37.69 ± 3.54 mm (range; 30.77–45.78 mm). Anteroposterior measurements obtained at the beginning and end of the medial cuneiform articular surface and at the terminal level of the dorsal surface were 18.01 ± 2.34 mm (range; 12.99–22.31 mm), 15.15 ± 2.15 mm (range; 11.28–20.46 mm) and 12.56 ± 1.62 mm (range; 8.29–16.25 mm), respectively. (χ^2^(2) = 108.036, *p* < 0.001). The mean depth measurements at these levels were recorded as 18.35 ± 3.01 mm (range; 12.71–27.60 mm), 25.24 ± 2.30 mm (range; 20.40–29.50 mm) and 22.52 ± 2.47 mm (range; 16.40–28.60 mm), respectively (χ^2^ (2) = 89.345, *p* < 0.001). The anteroposterior dimension of the lateral surface was 11.60 ± 1.54 mm (range; 8.74–15.13 mm) (Table 2). The depth of the talonavicular articular surface was 5.27 ± 0.92 mm (range; 3.03–7.17 mm) (Table 1).

**Table 2:** Width, Depth, and Anteroposterior Measurements (mm) of the Navicular Body

Parameter		Mean ± SD (mm)	10% – 90% percentile	Range	*p*
Mediolateral width		37.69 ± 3.54	33.44–43.31	30.77–45.78	-
AP measurements (of the dorsal surface)	at the beginning of the MCAS	18.01 ± 2.34	14.66–21.05	12.99–22.31	** <.001**
	at the end of the MCAS	15.15 ± 2.15	12.33–18.30	11.28–20.46	
	at the terminal level of the dorsal surface	12.56 ± 1.62	10.35–14.66	8.29–16.25	
Depth	at the beginning of the MCAS	18.35 ± 3.01	15.32–22.32	12.71–27.60	** <.001**
	at the end of the MCAS	25.24 ± 2.30	22.30–28.50	20.40–29.50	
	at the terminal level of the dorsal surface	22.52 ± 2.47	19.72–26.08	16.40–28.60	
AP dimension of the lateral surface		11.60 ± 1.54	9.56–13.35	8.74–15.13	-

*SD* standard deviation, *MCAS* medial cuneiform articular surface, *AP* anteroposterior

*p* values represent overall comparisons by Friedman test. Pairwise comparisons were performed using the Wilcoxon signed-rank test with Bonferroni correction, and all pairwise comparisons were statistically significant (*p* <.001)

## Discussion

Navicular fractures are surgically challenging because of the bone’s central role in talonavicular and naviculocuneiform biomechanics. Successful treatment requires anatomical reduction, restoration of talonavicular congruence, and preservation of medial column length congruence [8]. However, the complex three-dimensional morphology, limited vascularity, and broad articular surfaces of the navicular make implant placement technically demanding and may increase the risk of joint penetration, malreduction, and insufficient fixation [1]. Indeed, the AO surgical guidelines emphasise that preserving navicular morphology during dorsal plating and screw placement is critical for achieving surgical stability and maintaining joint congruence. However, the extant literature is primarily focused on the classification, surgical clinical outcomes and biomechanics of navicular fractures [1–3], and detailed osseous morphometric data that could directly guide implant placement remain limited.

The navicular tuberosity is surgically important, particularly in avulsion fractures, as it serves as the primary insertion site of the posterior tibialis tendon. In such fractures, conservative treatment or surgical fixation may be preferred depending on the fragment size, degree of displacement, and stability requirements; when surgery is indicated, lag screw fixation, suture anchor techniques, or transosseous/pull-through suture methods can be applied [8]. Consequently, the morphometric characteristics of the bone stock can be crucial in determining the diameter, length, and entry point of implants to be placed in the tuberosity region.

In the present study, the mean supero-inferior length of the tuberosity was 11.24 ± 1.26 mm, decreasing to 6.59 ± 1.28 mm at the proximal end and to as little as 2.90 mm in some specimens (Table 1). This suggests that bone stock is not uniform throughout the tuberosity and may be particularly limited in the proximal segment. Given that screw diameters utilised for navicular fixation have been reported to range from 2.0–3.5 mm in acute fracture treatment to 4.0 mm in selected fixation techniques [9–12], this regional narrowing of the tuberosity may represent an important anatomical constraint in implant size selection and screw trajectory planning. Moreover, the mean depth of the groove on the inferior surface was 2.05 mm, indicating a potential anatomical constraint that may increase the risk of extraosseous trajectory in interventions adjacent to the inferior border. Accordingly, avoiding placement of the entry point too close to the inferior border of the tuberosity may be important for maintaining an intraosseous trajectory. Additionally, the mean anteroposterior length of the tuberosity was 16.38 ± 2.43 mm, defining a limited osseous corridor for multiple-screw fixation. This distance should be considered when determining screw number, diameter, and inter-screw spacing in relation to the available bone stock.

**Fig. 2:**
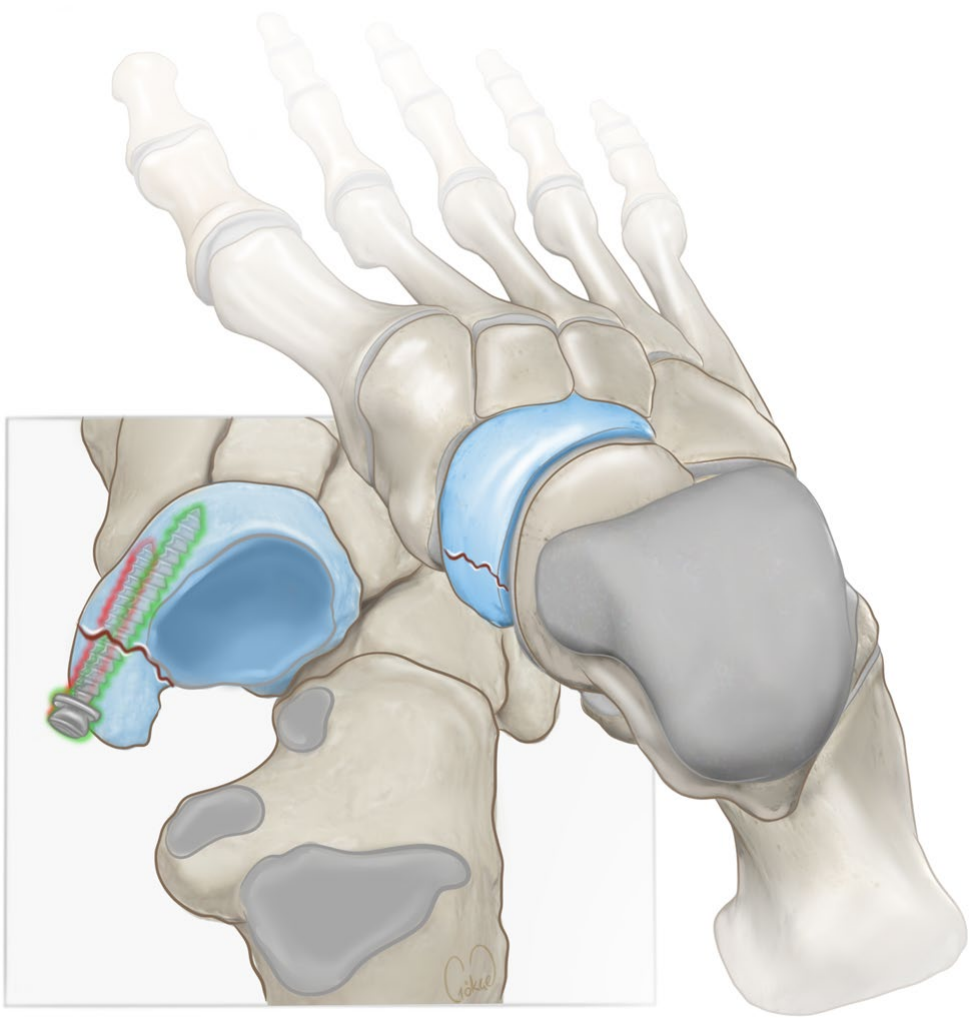
Schematic illustration of the distances from the midpoint of the navicular tuberosity to the medial cuneiform articular surface in relation to a tuberosity avulsion fracture, relevant to lag screw fixation planning. The ***neon red screw*** represents the shortest trajectory towards the medial column, whereas the ***neon green screw*** represents the longest trajectory

The distances from the midpoint of the tuberosity to the medial cuneiform articular surface were measured at approximately 22 mm and 30 mm at the nearest and farthest points (Table 1), respectively. These measurements provide a practical anatomical reference for planning screw length in fixation directed from the tuberosity towards the medial column. In tuberosity avulsion fractures treated with lag screws, the primary surgical objective is to achieve adequate stabilisation while avoiding articular penetration. Accordingly, these measurements may guide implant length selection and screw placement to reduce this risk (Fig. 2).

**Fig. 3:**
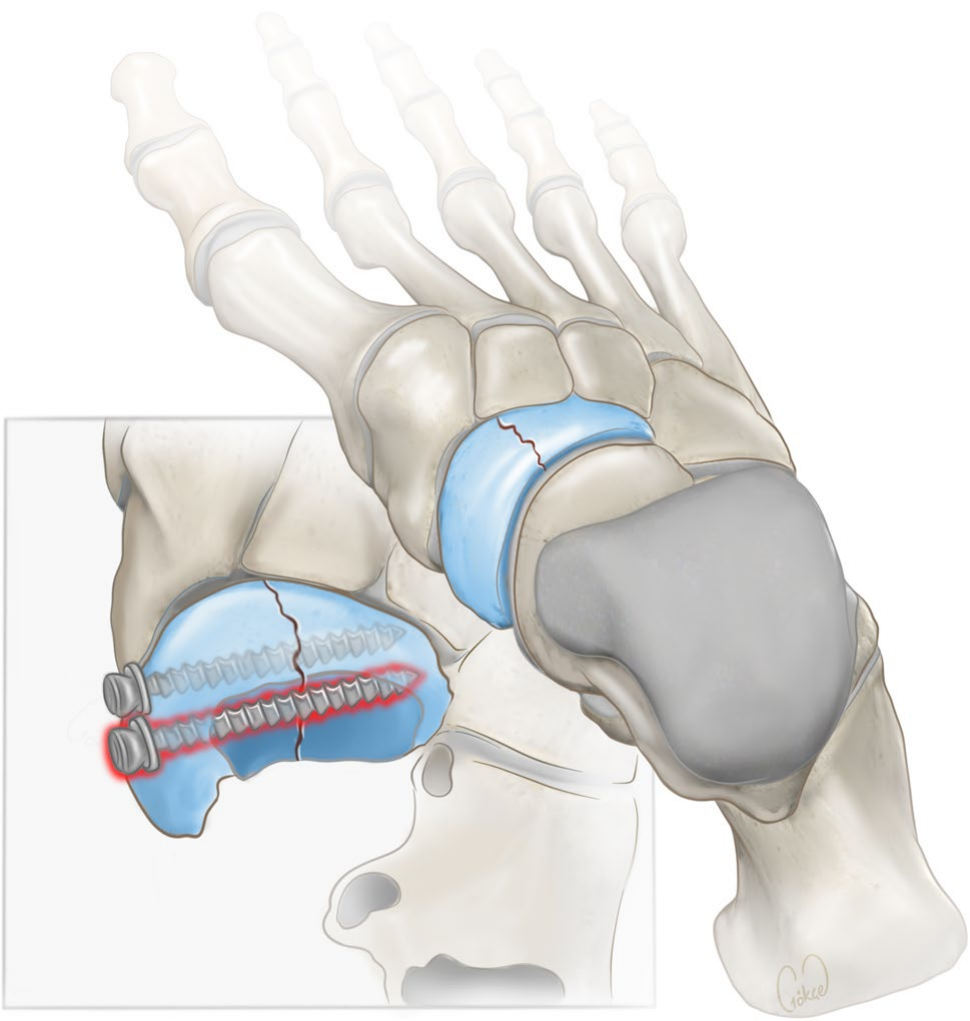
Schematic illustration of screw trajectories from the navicular tuberosity towards the lateral side in a typical simple navicular fracture. The ***neon red screw*** demonstrates potential articular penetration if the depth of the talonavicular joint surface is not considered

In addition to medial column-directed fixation, in screw placements directed laterally from the tuberosity region of the navicular bone, morphometric data regarding the depth of the talonavicular joint surface (5.27 ± 0.92 mm in this study) are clinically relevant for reducing the risk of articular penetration (Fig. 3). Consequently, not every laterally directed screw trajectory along the tuberosity may provide a safe intraosseous corridor, as the depth of the talonavicular joint surface may constrain the available trajectories and increase the risk of articular penetration. Furthermore, AO surgical principles emphasise that excessive compression applied during reduction has the potential to disrupt the physiological concavity of the talonavicular joint. In this study, the distribution of the concavity index (median 5.03; interquartile range 4.71–5.52; range 4.01–7.44) demonstrated significant morphological variations among the samples, ranging from pronounced concavity to relatively flatter configurations. These findings highlight that the talonavicular articular surface does not have a uniform concave morphology and that this variability should be considered during reduction and fixation.

**Fig. 4:**
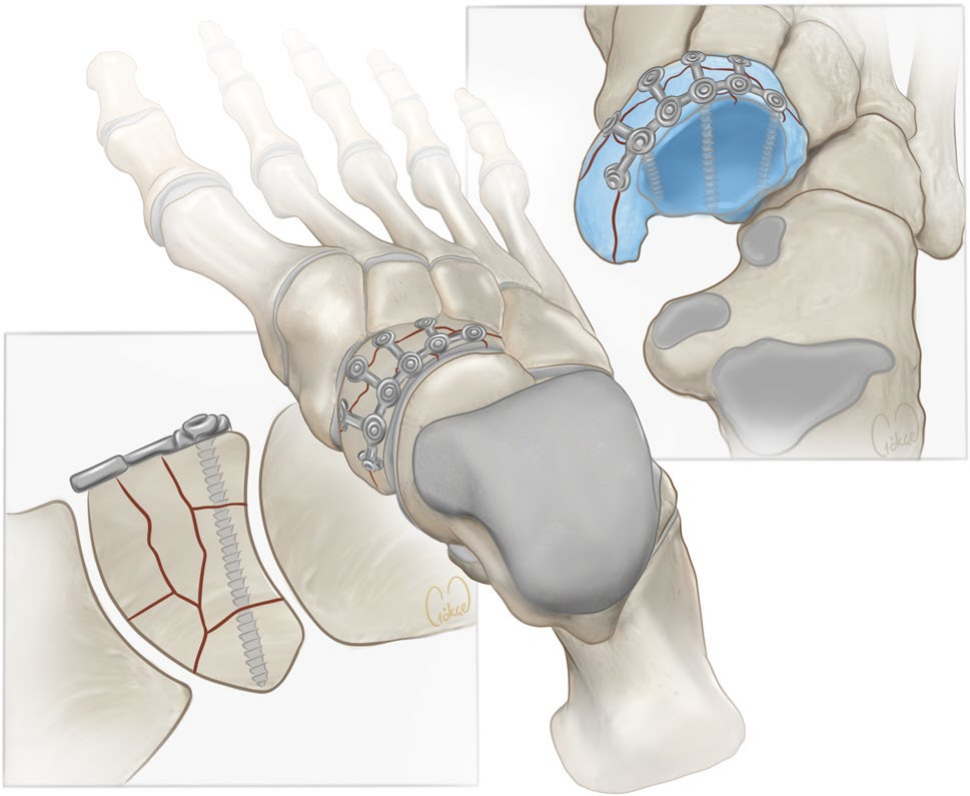
Schematic illustration of dorsal plate fixation for a complete articular navicular fracture, showing the use of different screw lengths according to level-specific osseous geometry to reduce the risk of cortical perforation

Beyond tuberosity-related fixation, navicular body dimensions are particularly important for dorsal plate placement and screw planning in displaced body fractures. ORIF is commonly used for displaced navicular body fractures. According to AO principles, interfragmentary lag screws are recommended for simple fractures, whereas plate-screw constructs or bridging techniques are preferred in comminuted or more complex patterns. The aim is to restore articular alignment, preserve medial column length, and achieve stable fixation. In the present study, the mean mediolateral width was 37.69 ± 3.54 mm. This dimension has been documented in the literature as 34.7 ± 5.6 mm (range; 26.0–45.0) and 40.4 ± 2.7 mm [13, 14], suggesting that our findings are consistent with established morphometric ranges and may provide a useful anatomical reference for dorsal fixation planning. More importantly, the significant variation in anteroposterior dimensions (Table 2, < 0.001 for all pairwise comparisons) across levels demonstrates that the available osseous corridor for dorsal plate placement is not constant along the navicular body. This narrowing, which becomes more pronounced in the distal segment, suggests that the fit of the plate to the bony surface and its distal placement may not be maintained in the same manner throughout dorsal plating. Therefore, plate contouring and distal positioning should be individualised according to the narrowing osseous anatomy of the region and planned within the available osseous corridor without approaching the articular surface. Additionally, the mean body depth measurements were approximately 18 mm, 25 mm, and 22 mm at these levels, respectively, and all pairwise comparisons were statistically significant (*p* < 0.001) (Table 2). Taken together, these findings indicate that the navicular exhibits a surgically relevant, non-uniform, and level-dependent osseous geometry. These differences are clinically relevant when planning screws through the navicular body, particularly for selecting appropriate screw length and avoiding cortical perforation (Fig. 4). Although a wide range of screw lengths is commercially available, the present data suggest that the same screw length may not be suitable for all regions of the navicular. Therefore, screws placed through the navicular body should not rely on a uniform screw length; rather, both screw length and trajectory should be determined according to the local osseous anatomy at each level to reduce the risk of cortical perforation.

Furthermore, these findings suggest that, in terms of navicular plate design, screw-hole placement, screw angulation, and plate geometry should be planned according to the regional morphometry of the bone. Screws placed in regions adjacent to the talonavicular articular surface and around the tuberosity may benefit from the use of variable-angle or controlled-angulation systems rather than fixed-angle systems, because the risk of joint penetration, cortical perforation, and extraosseous screw placement may be higher in these regions. In contrast, the portion corresponding to the medial cuneiform articular surface, where the anteroposterior dimension of the navicular body is widest, may provide a more suitable osseous corridor for double-row or multiple screw configurations. Therefore, positioning the second or third row of screw holes on the plate so that they correspond to this wider medial region rather than the narrowing lateral segment may represent a safer design approach. The lateral/distal narrowing and non-uniform geometry of the navicular body suggest that completely rectangular or standard mesh plates may not always conform adequately to the navicular anatomy. Although such plates can be contoured, excessive implant bending may not always result in optimal anatomical conformity and may potentially affect the mechanical properties of the implant [15]. Furthermore, the level-dependent variation in body depth also supports the planning of region-specific screw length options in navicular plate systems. Rather than recommending the same screw length range for every hole, different screw length options or hole-based screw length guides may be developed according to the proximal, middle, and distal segments in order to reduce the risk of cortical perforation. Consequently, navicular plate systems should not rely solely on medium/large size distinctions based on mediolateral length, but should also include more anatomical and region-specific sizing options that take into account the distal narrowing pattern, anteroposterior width, and regional depth parameters. In this context, the present data may contribute to the development of anatomically compatible fixation implants requiring less intraoperative modification.

In addition to fixation implant design, these morphometric data may also be relevant to reconstructive implant planning. The current literature on navicular bone prosthetic reconstruction is limited to selected case reports and small reconstructive series using patient-specific 3D-printed implants, most often in advanced osteonecrosis, critical bone loss, marked deformity, or when conventional reconstruction is insufficient [16–19]. While the short- and medium-term results reported to date are encouraging [17, 18], the available evidence remains limited. In this setting, the level-specific morphometric data presented here may be relevant not only for implant placement in fracture fixation but also for defining regional anatomical variability that should be considered in personalised reconstructive planning. The decrease in anteroposterior dimension and the non-uniform distribution of depth along the body reveal that the navicular bone does not possess a uniform bone geometry that should be taken into consideration.

Directly comparable literature for the parameters assessed in this study is limited, making one-to-one numerical comparisons difficult. Most available studies have focused on the classification, clinical outcomes, and surgical management of navicular fractures [1–4, 6], whereas morphometric investigations have mainly described overall bone dimensions or anatomical variations [20, 21]. By contrast, level-specific osseous morphometric data with direct relevance to implant placement remain scarce. The present study addresses this gap by providing quantitative anatomical reference data relevant to fixation planning.

## Conclusions

The present study demonstrated level-specific morphometric differences in the navicular tuberosity and body that are relevant to fracture fixation. These quantitative data provide anatomical reference values that may assist implant selection, estimation of safer fixation corridors, and preoperative assessment of the risk of cortical perforation or articular penetration. The findings support preoperative planning by highlighting regional osseous variability that should be considered during navicular fixation.

## Limitations

There are several limitations to this study that should be taken into consideration. Firstly, the measurements were performed on dry bones with unknown age and sex, precluding analysis of possible demographic effects on morphometry. Secondly, due to the absence of articular cartilage, soft tissues, and fracture-specific deformation, it cannot be assumed that the measured osseous dimensions accurately reflect the surgical corridors encountered intraoperatively. Thirdly, because it was not possible to ascertain whether the right and left bones belonged to the same individuals, side-to-side differences could not be interpreted as true biological lateralisation. Finally, the present data were not correlated with radiographic planning, intraoperative fluoroscopy, implant-specific biomechanical testing, or clinical outcomes. Accordingly, the findings should be interpreted as anatomical reference values for surgical planning rather than as definitive operative rules.

## Data Availability

No datasets were generated or analysed during the current study.
